# A critical systematic review assessing undergraduate neurology pipeline programs

**DOI:** 10.3389/fmed.2023.1281620

**Published:** 2023-11-21

**Authors:** Mia T. Minen, Ramisha Aymon, Ishah Yusaf, Khushalee Oza, Jane Ekhtman, Aarti Katara, Naomi Lebowitz, Caitlin Plovnick

**Affiliations:** ^1^Department of Neurology, NYU Langone Health, New York, NY, United States; ^2^The City College of New York, New York, NY, United States; ^3^Barnard College of Columbia University, New York, NY, United States; ^4^Medical Library, NYU Grossman School of Medicine, New York, NY, United States

**Keywords:** undergraduate, education, neurology, neuroscience, pipeline programs

## Abstract

**Background:**

Although current programs exist to encourage undergraduate interest in neuroscience and neurology, few students go on to pursue a career in neurology. Thus, there is a need for more neurologists in the US. To assess undergraduate pipeline programs and their goals of garnering interest and knowledge of neurology, we systematically reviewed available literature on existing undergraduate neurology pipeline programs.

**Methods:**

A medical librarian conducted an electronic database search of PubMed, EMBASE, PsycINFO, Education Source, and ERIC based on a search strategy developed with a team of undergraduates and a neurologist. Of the 2,852 articles screened, 33 met the systematic review criteria and were evaluated based on the type and goal of the pipeline program, its delivery, and efficacy.

**Results:**

The 33 programs were classified into subtypes of pipeline programs, with focuses ranging from student-led projects to early clinical research opportunities. All programs were found to be successful in attracting student interest in neurology, providing exposure to relevant opportunities, and classroom enrichment.

**Discussion:**

The existing literature shows that neurology pipeline programs successfully inspire interest in a career in neurology among undergraduate students. These programs are valuable supplements to undergraduate neuroscience curricula and instrumental in introducing students to various fields.

## Introduction

Over 7,208 students major in neuroscience in college (1), and nearly one-fifth of them express interest in going to medical school. However, under 3 % of pre-medical students matriculating into medical school indicate an interest in pursuing a career in neurology. Thus, a disconnect appears between this expressed interest in neuroscience among undergraduates and the long-term pursuit of the field (2). This dwindling interest among undergraduate students is oftentimes attributed to limited clinical experience, lack of guidance and support from mentors, and insufficient knowledge about neuroscience that motivates them to pursue such a field of study (3). Additionally, the COVID-19 pandemic has led to increased concern among neurology residents on the future of neurological patient management, care, and research, which could translate to unease about the profession at the undergraduate level (4).

Early exposure to neuroscience-based training programs and research opportunities has been proven to bolster students’ interest in learning about neuroscience (5). However, such programs for undergraduate students appear inaccessible to a wide student population, making integration into the classroom and related settings imperative to create a more adaptable way of learning (6). A pipeline program, described as a structured educational pathway that guides students from an early stage through various stages of education and training toward a specific career, plays a vital role in ensuring broader accessibility to certain career paths. Expanding neuroscience curricula with diverse programs or research opportunities may not only make neuroscience more interdisciplinary for students but also garner interest among underrepresented minority (URM) students and better inform them of related careers to pursue in neuroscience (7).

We conducted a systematic review of the literature on past and current undergraduate neurology pipeline programs to better understand their intent in garnering student interest in neuroscience. Our research question was: What neurology pipeline programs have been designed and implemented to attract undergraduate students into neurology? We also wanted to further analyze program designers, delivery methods, target audience (including any programs for URM students due to a shortage of URM neurologists), and success measurements.

## Methods

We conducted a systematic review to identify existing neurology pipeline programs using the Population, Intervention, Control, and Outcome (PICO) framework to strategize and develop our research question. We limited our population for this systematic review to undergraduate students; studies of programs that focused on populations of K-12 students were evaluated in a separate review. Interventions refer to the implementation of neurology pipeline programs intended to impart knowledge about neuroscience to students. We also measured the outcomes of the programs or the success with which they achieved such goals.

Under the guidance of a neurologist (MTM), a team of six undergraduate research assistants (AK, IY, JE, KO, NL, and RA) iteratively created a list of keywords related to neurology education and career pipelines, which a medical librarian (CP) then expanded and refined. The librarian searched PubMed, Embase, and PsycINFO via the Ovid platform and Education Source and ERIC via the EBSCO platform for articles describing neurology pipeline programs. For our study, this was defined as: any initiative that introduced, educated, or imparted students with the knowledge and an interest in neurology that may inspire them to explore these interests to potentially pursuing the field. Each search strategy included a combination of keywords and controlled vocabulary appropriate to each database. The complete search details can be found in Appendix A. The strategy was registered via the Open Science Framework (OSF) with the registration doi: 10.17605/OSF.IO/2G8CN.

**Figure 1:**
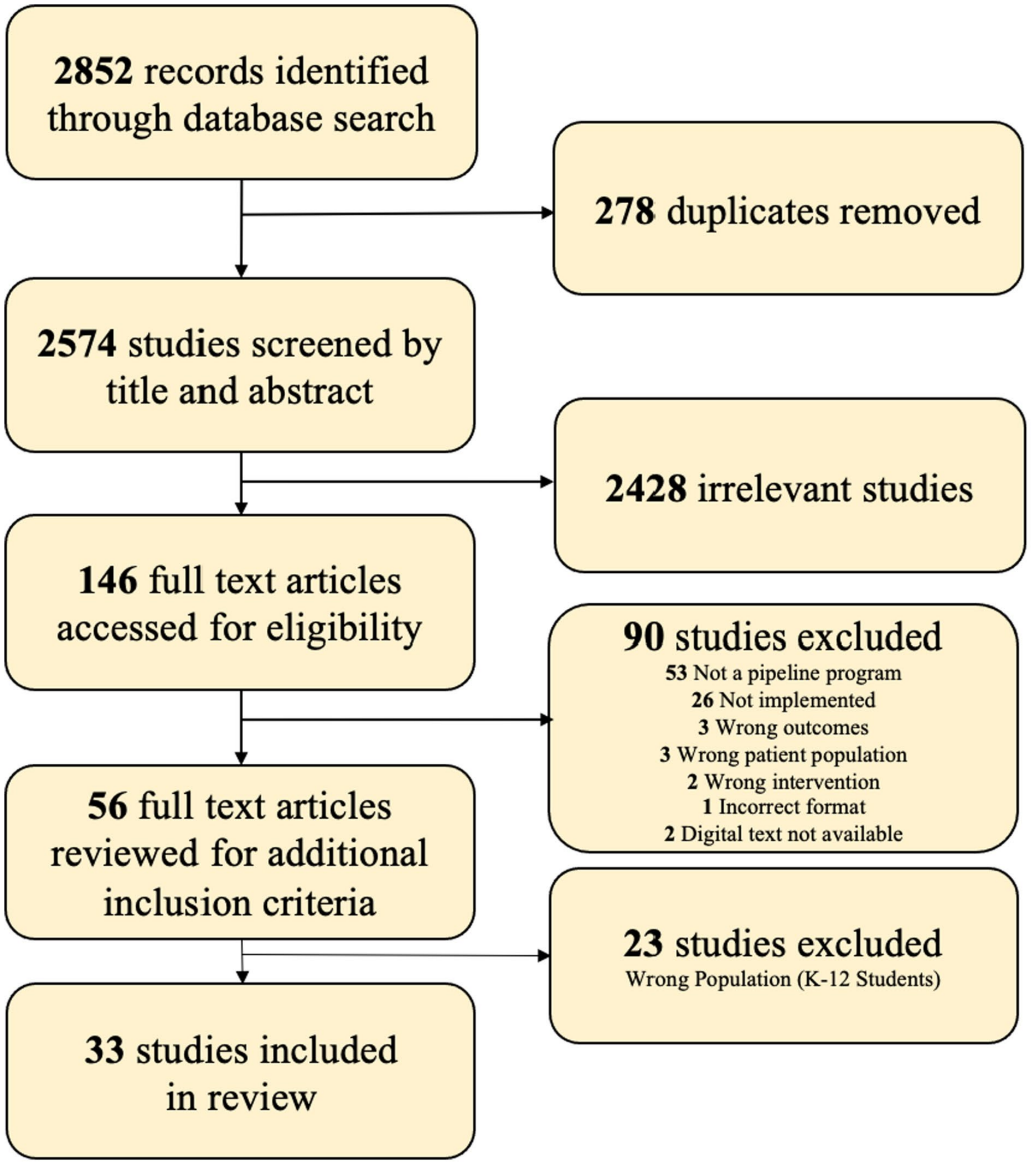
Flow diagram.

The search was conducted on July 5, 2022, and was not limited by the language or year the article was published. The resulting citations and abstracts were put into Covidence software. As shown in the PRISMA (Preferred Reporting Items for Systematic Reviews and Meta-Analyses) diagram (Figure 1), 278 duplicate studies were removed, and 2,574 studies were screened for inclusion by the six reviewers (AK, IY, JE, KO, NL, and RA) based on the following inclusion criteria: (1) The program must be related to neuroscience or neurology, (2) The population of participants was limited to students in either K-12 education or undergraduate college students, and (3) The program had to have been implemented and provided specified outcomes. Before the Covidence screening, the medical librarian (CP) met with the reviewers to review the screening process and to ensure fidelity. Two different reviewers independently screened and voted upon each citation, and disagreements were resolved through discussion, resulting in a final vote. 146 studies were selected for full-text review, with 56 ultimately meeting the inclusion criteria after two rounds of in-depth independent screening. These 56 studies were then differentiated by targeted age groups: K-12 and undergraduate students. Of these, 20 were relevant to the K-12 age group, 28 were relevant to undergraduates, and 8 described programs that included participants in both target groups. In the mixed group, the reviewers determined which age groups benefited more directly. Ultimately 33 studies were included for this review, 28 exclusively targeted to undergraduate students, and 5 from the mixed group section of articles. A summary of the included programs is included in Table 1.

**Table 1:** Summary of included programs.

Title, First Author, Year, Journal	Program type	Program goals	Participants	Program duration	Program measures	Program outcomes
Neuroscience and Sustainability: An Online Module on “Environmental Neuroscience” (8) *The Journal of Undergraduate Neuroscience Education (JUNE)* Year Published: 2018	Neuroscience concepts were incorporated into a mid-level biopsychology course through an online “Environmental Neuroscience” module	To enhance students’ understanding of neuroscience and how it can contribute to both sustainability efforts and other scientific disciplines (e.g.: environmental psych)	Participants: 26 students	The module was conducted over the course of the semester, with an “Environmental Stewardship Portfolio” due at the end of the term. Students wrote reflection papers based on five articles of their choosing, each due every 2–3 weeks. Most of the work was done outside of class, meaning the module did not take up class time.	Students’ papers were given critiques for revisions, completed an anonymous evaluation of the module, and took a knowledge test with results compared to a control group (n = 27) of students who did not take the online module but the same course.	Students mostly showed significant increase in knowledge and awareness of neuroscience and how it connects to other disciplines of science, like environmental issues, and also scored higher than the control group on the knowledge assessment (median score of 67.8, SD of 16.9).
Integrating Brain Science into Health Studies: An Interdisciplinary Course in Contemplative Neuroscience and Yoga (9) *The Journal of Undergraduate Neuroscience Education (JUNE)* Year Published: 2011	Undergraduate health class combining contemplative neuroscience with yoga practice to introduce students who might otherwise avoid taking explicit neuroscience classes to elements of neuroscience in an integrative and accessible manner	Engage a broader student population in neuroscience learning, beyond those already interested in science, by relating neuroscience to health concepts through the interdisciplinary field of contemplative neuroscience	Participants: 80 students between fall 2012 and spring 2015 in sections of 10–16 students	Semester-long course that met for 1 h and 45 min once a week with ~50–55 min of lecture/discussion followed by yoga practice relevant to the contemplative neuroscience topics previously discussed; required readings were accessible to a lay audience; students were required to complete a weekly journal and one final exam	One cohort of 12 students was administered a 10-item multiple choice neuroscience pre/posttest; at the end of the semester, students complete a course rating assessing their progress in course objectives on a Likert scale	Significant increase in scores on neuroscience knowledge posttest; average student course ratings indicated that students had more positive feelings about the field of neuroscience after taking the course
Reading the Brain: An Interdisciplinary First-Year Seminar on the Intersection of Neuroscience, Literature, and Popular Culture (10) *The Journal of Undergraduate Neuroscience Education (JUNE)* Year Published: 2021	Interdisciplinary 1st-year seminar that is taught on the intersection of neuroscience, literature, films, plays and advertisement + ways that brain science can help people understand why and how we read taught by cognitive neuroscientist and literary scholar	To highlight benefit of using non-technical sources to introduce students to scientific concepts and value of teaching collaboratively across disciplinary boundaries. Engaging students in cross-disciplinary thinking and helping neuroscience students grasp difficult topics (achieve better learning outcomes)	Participants: 32 students (for 2 years)	Course was a part of first-year seminar program that is limited to small # of students. Course was taught in 2 subsequent academic years. Class met weekly for two 75-min periods. Used discussion format course was divided into 8 segments	- assessed the degree to which the student’s writing has improved over the semester (compared results on students’ 1st analytical paper to performance on students’ 2nd analytical paper), - assessed analytical paper over 3 metrics: literary analysis, brain literacy and interdisciplinary to see impact - administered mid-semester and end-of semester evaluation to assess degree of which the course was successfully implemented according to students	Students had positive view on course content and course interdisciplinary writing improved over the course and ability to make interdisciplinary connections in both class and written assignments. 68% (11) of students went on to declare majors, 11% of students declared minors in STEM disciplines
Drugs, the brain, and behavior: a graduate student-run comprehensive course in neuroscience (12) *The Journal of Undergraduate Neuroscience Education (JUNE)* Year Published: 2021	2 semester upper-level course that employs an interdisciplinary approach to normal and altered nervous system function; aimed toward both undergraduates and PhD students	expose students to neuroscience + knowledge in neurological and neuropsychiatric illness; promote scientific literacy in undergraduates and provide pedagogical experience for PhD students	Participants: 240 students in their second/third year	run and taught by Ph.D. students in the Interdisciplinary program in Neuroscience at Georgetown University/24 students	students enrolled in fall semester filled out a questionnaire after the midterm (agreements with 9 statements of proficiency in topics taught) – asked to estimate agreement prior to start of the course and at the time of the midterm compared final and midterm exam scores	in the fall semester, students showed significant improvement (21%) in raw scores between the midterm and final examinations, all students rated themselves at a higher level in their proficiency of the topics taught compared to the beginning of the course
Social neuroscience at the college of saint rose: the art of team teaching in emerging areas of psychological science (13) *The Journal of Undergraduate Neuroscience Education (JUNE)* Year Published: 2010	a new undergraduate level course for neuroscience that merges psychology and neuroscience	to develop an effective team-taught course in social neuroscience at the undergraduate level, provide undergraduate students with access to new, developing content merging social psychology and neuroscience	Participants: 15 students	traditional lecture-style course with seminar-style course combination, students were assigned readings from text and one student was deemed discussion leader for that text, three exams delivered throughout the term, students required to develop research proposal and present it to the class	students were given a anonymous questionnaire and demographic survey at the end of the course, two-tailed one sample t-tests used to analyze data	students reported gaining a better understanding of the material through team teaching vs. single teacher teaching, students reported enjoying taking the newly designed course
Using case studies as a semester-long tool to teach neuroanatomy and structure–function relationships to undergraduates (14) *The Journal of Undergraduate Neuroscience Education (JUNE)* Year Published: 2010	Model-based program having students work together with case studies as a tool to teach brain structure–function relationships.	Implement basic and applied neuroanatomy with the fundamentals of structural organization of the nervous system.	Participants: 50 undergraduates	Students met three days a week over the 14-week semester and had seven days devoted to presentations and discussions of assigned case studies.	Program was evaluated from quality of student presentations, assessment of knowledge, discussion questions and level of engagement, as well as peer feedback.	85% of students reported positive feedback in using the case studies to learn concepts, as well as more peer engagement. One criticism was feeling more time was needed to learn concepts, feeling some were too “condensed.”
Professor Eric Cannot See: A Project-Based Learning Case for Neurobiology Students (15) *The Journal of Undergraduate Neuroscience Education (JUNE)* Year Published: 2016	Case study incorporated in an upper-level undergraduate course	Focuses on teaching to critically evaluate primary literature and analyze symptoms and treatment of relevant case-study scenario.	N/A	Taught in a Neurobiology of Disease course, case had five scenes and an in-class worksheet, unit was taught in six 75-min class periods.	Tracked progress with in-class and homework assignments, students were asked to thoughtfully identify learning objectives and took a final exam.	Overall class average from unit exam was 83.9%, and some provided positive feedback in wide range of material taught from the case-study.
Using Case Studies to Promote Student Engagement in Primary Literature Data Analysis and Evaluation (16) *The Journal of Undergraduate Neuroscience Education (JUNE)* Year Published: 2017	Lesson plan for incorporating literature-based neurology case studies into cellular and molecular neuroscience course for upper-level undergraduates.	Increase student engagement with primary/basic neuroscience literature, especially among students interested in clinical healthcare careers	Participants: 29 students	Students worked in small groups to discuss case studies with Pretzel Syndrome and ALS and answered questions; whole class reviewed in larger discussions throughout course.	Assessed learning with take-home exams and gave individual pass/fail grades; qualitative student evaluation of lesson (verbal feedback) and quantitative (Likert scale) before and after lessons.	Students appreciated clinical/real-world applications; increased confidence in understanding neurodevelopment, cellular/molecular neuroscience, and diseases and drugs used in treatments.
The Design, Implementation, and Assessment of an Undergraduate Neurobiology Course using a Project-Based Approach (6) *The Journal of Undergraduate Neuroscience Education (JUNE)* Year Published: 2018	Project-based approach consisting of student-led work, team-authored research papers, and poster presentations to improve understanding of neuroscience.	Better engage students in upper-level neurobiology course and promote positive attitudes in confidence with material.	Participants: 37 upper-level undergraduate students	Students met 2x a week during 15-week semester; each class meeting was 110 min long and taught by 1 instructor. 3 main project themes (neurotoxins, spinal cord injury and learning and memory).	Pre- and post-tests given to students to measure content knowledge, students completed pre- and post-surveys during first and last week of class to measure confidence in neuroscience and attitudes working in team projects.	Greater performance on the post-test compared to the pretest, by end of course students reported significantly higher confidence in neuroscience knowledge.
‘Without A Key’: A Classroom Case Study (17) *The Journal of Undergraduate Neuroscience Education (JUNE)* Year Published: 2019	Case study using a narrative focused on Locked-in syndrome to engage upper-level undergraduates with neuroanatomy, clinical neuroscience and brain computer technology.	Allow students to work collaboratively to apply their analytical and course-related skills in an open-ended space to extended scientific skill development.	Participants: 25 students	Students worked in small groups in upper-level Cognitive Neuroscience course with both lecture and lab components. Case is preceded by brief review lecture and implementation takes 90 min. Class session	students provided anonymous feedback online about the case, 8 feedback questions assess the students’ qualitative experiences rating on a scale based on the goals of the program (specific content questions and general interest)	96% of participants agreed or strongly agree that exercise was “engaging and enjoyable,” 83% agreed it was more interactive than a traditional lectures, and 96% agreed that the case studies increased their knowledge of the topic and syndrome.
Effective Use of Student-Created Case Studies as Assessment in an Undergraduate Neuroscience Course (18) *The Journal of Undergraduate Neuroscience Education (JUNE)* Year Published: 2021	Students made case-studies used as an assessment tool for an online neurovirology module in a neuroanatomy and physiology course	improving student performance and increasing students’ knowledge and confidence in neuroscience	Participants: 24 students	Administered over zoom during weekly 3-h lab period for 4 weeks (week 3–4 used for case study assignment) students used writing practice to write their case studies (graded), students exchanged case studies with their peers and taught/completed case studies with their peers as a low-stakes assessment	Students used a Likert scale to self-report their knowledge of content learning objectives and confidence in skills learning objective after completion of the module and the case-study assignment compared students grades of 1st and final draft of case study.	Grades increased compared from first exam to the final, supporting that interactive writing practices and low-stakes exams can improve performance. Students reported that writing, teaching and completing a neurovirology case-study all increased knowledge – writing ranked higher than teaching or completing.
Learning Neuroscience: An Interactive Case-Based Online Network (ICON) (19) *Journal of Science Education & Technology* Year Published: 2002	ICON, interactive case-based online network, that is introduced to undergraduate curriculum to bring a different focus on reshaping their thinking in neuroscience	Enhance students’ approach to problems, solutions, and insights in neuroscience	Participants: 12 undergraduates	Applied in Harvard Faculty of Arts and Science curriculum as an honors elective (for 1 semester), students met face to face for 2 h 1x week in two tutorial groups of 6 students each.	letter grades for the tutorials were assigned according to online participation, contributions to discussions, written case summaries, and final examination - also had students and faculty submit an evaluation of the program as a learning tool	full participation in all modules; thoughtful analysis of the each other’s scientific reasoning in the case studies, weekly face to face meetings were lively and conversation picked up from the previous meetings (ICON supported the active participation of the faculty and students’ learning)
Online Teaching Tool Simplifies Faculty Use of Multimedia and Improves Student Interest and Knowledge in Science (20) *CBE – Life Sciences Education* Year Published: 2011	Developed Online Multimedia Teaching Tool (OMTT) in Neuroscience for faculty to use to help deliver online resources in a similar format used in their lectures. Students had unlimited online access for self-exploration on the topic of neuroscience	Developed to help increase student knowledge and interest in neuroscience + simplify instructor adaptation by giving a lecture-based method of digital curriculum	Student participants – 221 Faculty – 5	Supplemented existing curricula in multiple courses, −1st Beta phase released on Parkinson’s disease, 2nd phase released OMTT chapters on Ion channels, Synapses. 3rd phase released Functional Neuroanatomy and Biogenic Amines – developed over 2 years which students and faculty had access to, giving instructional login at the beginning of the semester and results compared at the end of the course	Comparing outcomes for students who volunteered to participate in the program to a control group of students in that class who did not choose to register for OMTT for the course. Conducted survey of user opinion on use of OMTT. Also compared whether students who used OMTT had better performance on traditional in-class forms for assessment.	OMTT used at USC during Fall Semester of 2009 showed registered students did significantly better when instructor used OMTT in lectures and when exam covered material in the OMTT, OMTT in neuroscience viewed positively by students across all 4 courses, also felt instructors’ use of OMTT in class helped them understand material better and made them want to self-explore OMTT in their free time
The Lateralizer: A Tool for Students to Explore the Divided Brain (21) *Advances in Physiology Education* Year Published: 2012	A tool that implements the divided visual field (DVF) technique, introduced as a problem-based learning module in a research methods course, students designed DVF experiments.	To help students explore the differences between the left and right cerebral hemispheres	Participants: 240 upper-level undergraduates	Program was introduced during a 2-week module in a large research methods course halfway into the semester, large weekly lecture (50 min), 2 h laboratory classes a week	Research reports, multiple-choice questionnaires about the lateralization of cognitive function, the anatomy of the brain, and the spatial frequency hypothesis to test student learning outcomes, pretest and posttest.	significant improvement in scores from pretest to posttest, students gained a new knowledge of brain anatomy and connectivity, project added to students’ research skills
The Open PicoAmp: an open-source planar lipid bilayer amplifier for hands-on learning of neuroscience (22) *PLOS ONE* Year Published: 2014	An open-source lipid bilayer amplifier was developed for use in neuroscience introductory courses.	Hands-on approach to learning neuroscience to teach about different electrical properties of the cell membrane.	N/A	It was about a 4-h laboratory session. Usually, one group of two students is assigned per bilayer setup. To allow the session to run smoothly and maximize the interactions with the students, one instructor for two groups of students is an optimal configuration. Lab sessions at our institution are run with ten bilayer setups working simultaneously.	Program was studied by the adjuncts instructors and older students which have been previously trained.	In a typical four hours session, all students were able to obtain the lipid bilayer formation and study its electrical properties. Also, a majority of attendees could actually succeed to observe the single channel currents. This was a good performance, as it is a rather delicate experiment and for many students this was their first experience in such a laboratory environment.
Neurogaming Technology Meets Neuroscience Education: A Cost-Effective, Scalable, and Highly Portable Undergraduate Teaching Laboratory for Neuroscience (23) *The Journal of Undergraduate Neuroscience Education (JUNE)* Year Published: 2017	A teaching lab with equipment to incorporate into an introductory neuroscience curriculum. The devices used to EEG machines for the students to use as a research tool and enhance student’s’ introduction into neuroscience	Use cost-effective tools and implement a teaching lab to be able to expose students to laboratory/research experience	Participants: 268 undergraduates, but only 150 students fully completed the evaluation survey	EEG headsets and software were incorporated to students’ lab sessions over the course of their semester, with each lab session about 1-h long	Students took in-class surveys with a 5-point Likert scale with responses consolidated into three findings: enjoyment, usefulness, and improvement on understanding; and their completed labs were looked into	Students have shown that the EEG-incorporated labs were enjoyable and increased their knowledge in neuroscience concepts and EEG, as well as provided them firsthand research experience.
Using A Contrast Illusion to Teach Principles of Neural Processing (24) *The Journal of Undergraduate Neuroscience Education (JUNE)* Year Published: 2018	an interactive laboratory exercise to challenge undergrads to use observational measurements of a visual contrast illusion to study neural activity	to understand the effectiveness of this active learning exercise in increasing student understanding of how perception is shaped by neural circuits in the retina	Participants: 112 students.	Students conducted simple psychophysical experiments to measure thresholds for detecting illusory spots under various conditions and described their results in a laboratory assignment.	in a pre and post survey, students’ confidence and practical understanding of 3 main concepts neural processing were evaluated on a Likert Scale.	students had significant improvements in content knowledge and increased confidence in ability to demonstrate understanding after the lab. Students had overall positive attitudes toward the lab.
Hands-on Undergraduate Experiences Using Low-Cost Electroencephalography (EEG) Devices (25) *The Journal of Undergraduate Neuroscience Education (JUNE)* Year Published: 2019	Implementing EEG through MUSE headbands.	To determine whether or not EEG can help students learn better about their brain	Participants: 26 upper-level undergraduate students	Students utilized EEG in classrooms to see how the brain functioned while doing several different brain exercises. This consisted of trying new tastes and answering different questions.	Students reflected on their own EEG data and filled out surveys to determine whether or not the implementation of EEG had an effect on understanding the brain.	Students felt more motivated to work on their current tasks and projects due to the EEG. Many students expressed enthusiasm and excitement for learning more.
Signal: A Neurotransmission Board Game (26) *The Journal of Undergraduate Neuroscience Education (JUNE)* Year Published: 2021	use of a board game designed to test students’ understanding of core concepts of core concepts covered in introductory neuroscience courses - action potentials and synaptic transmission	game-based learning serves an effective and engaging tool to review fundamentals of neurotransmission in science courses - allowed students to develop skills and gained a greater understanding of neuroscience	Participants: 36 students (38 played the game but 2 opted out)	participants were undergraduate students enrolled in “Fundamentals of Neuroscience” course; students played signal during week 13 of semester an in-class activity in groups 4–5 players - carried out in 2 sections of course in 1 semester	After completing the board game, students asked to complete survey on their impressions of the exercise (rated their agreement to statements)	80.5% (n = 24) agreed or strongly agree (n = 5) that the game helped them assess their knowledge and understanding of action potential and 83.3% (n = 22) + (n = 8) agreed that it helped their understanding of synaptic transmission. 30 students agreed that it allowed to develop communication skills as they talked to their peers about scientific concepts
Evaluation of an undergraduate neuroscience research program at the University of Kentucky (7) *The Journal of Undergraduate Neuroscience Education (JUNE)* Year Published: 2003	neuroscience summer research experience	*Expose undergraduate students to various graduate careers, inform them of neuroscience opportunities, increase URM students, give them research project of their own	Participants: 80 students	throughout the summer in 5 different components - 1. neuroscience course, 2. independent research projects, 3. lunchtime seminars, 4. summer symposium, 5. oral presentations at the end of the summer	annual end of summer survey	participation in this program may have strengthened students’ applications to graduate schools, high satisfaction with the material students were exposed to, successfully increased URM in neuroscience research, participation in program increases chances students will apply to graduate school
Partnerships in Neuroscience Research Between Small Colleges and Large Institutions: A Case Study (4) *The Journal of Undergraduate Neuroscience Education (JUNE)* Year Published: 2018	Neuroscience internship program for students (ideally 2nd or 3rd years) at Stonehill College to participate in either the Basic Research or Clinical Research division of the Laboratory of Neuroscience in the Department of Psychiatry, at Harvard Medical School	Goal is to provide students in smaller colleges a program within a larger institution with resources to enrich their curricula and extracurricular experience; helps to give students an experience they can utilize when applying to graduate programs	Participants: 35 total interns have completed this program and went onto graduate programs; 25 responded to a survey done by the authors	An intern would then work 8–12 h/week during the school year, but then increase their workload to full time for 10 weeks (35 h/week) during the summer typically through the Stonehill Undergraduate Research Experience	Program studied based on student performance and completion of the program. Survey was done comparing the interns’ initial goals once leaving the program versus actual outcomes on where they are after college. Also largely studied on student feedback and impressions	Many stayed consistent with where they wanted to go versus where they ended up, specifically with doctoral programs (i.e.: medical school) with ~13. Many more students actually went into research with masters or PhD, with not many having that as an initial goal (2–3 responded as a goal, 8–10 responded as an outcome)
Integrating Research into the Undergraduate Curriculum: 1. Early Research Experiences and Training (27) *The Journal of Undergraduate Neuroscience Education (JUNE)* Year Published: 2020	This article expands upon the information presented in workshop discussions, focusing on ways to promote early research opportunities.	To figure out different benefits and challenges of implementing research in the undergrad experience	N/A	Studied different articles and information from workshops to sort out details of what is beneficial or not for students.	Program was studied based on how students from different backgrounds (experienced or non-experienced) were able to perform in their research.	Found that it is beneficial to support students as they discover science, grow in confidence and competence, and move toward a future that will be well served by a diverse group of thoughtful, curious, well-prepared individuals as they are able to enter the workforce.
Integrating Research into the Undergraduate Curriculum: 3. Research Training in the Upper-level Neuroscience Curriculum (28) *The Journal of Undergraduate Neuroscience Education (JUNE)* Year Published: 2020	This article expands upon the information presented in workshop discussions, focusing on ways to promote early research opportunities.	To figure out different benefits and challenges of implementing research in the undergrad experience	N/A	Studied different articles and information from workshops to sort out details of what is beneficial or not for students.	Program was studied based on how students from different backgrounds (experienced or non-experienced) were able to perform in their research.	Found that it is beneficial to support students as they discover science, grow in confidence and competence, and move toward a future that will be well served by a diverse group of thoughtful, curious, well-prepared individuals as they are able to enter the workforce.
Building the Neurology Pipeline with Undergraduate Students in Research and Clinical Practice (29) *American Academy of Neurology* Year Published: 2021	Article studies different logistics, potential benefits and challenges on implementing undergrad neuroscience clinical research.	This article discusses how working with undergraduate students represents a unique opportunity to expose students to neurology through research and clinical care while also generating many beneficial experiences for the student and the neurologist. We outline potential benefits, and logistical considerations, involved in working with undergraduates.	Participants: 50	Various different articles and data collected from previous research was studied to determine the different implications of clinical neuroscience.	Various different articles and data collected from previous research was studied to determine the different implications of clinical neuroscience.	We incorporated undergraduate student perspectives regarding the impact of their experiences on their personal and professional lives. In total, we received reflections (n = 32), emails (n = 4), applications for opportunities (n = 7), and a program feedback survey (n = 1). In a process considered exempt by the Wake Forest Baptist Institutional Review Board, we qualitatively analyzed these data into themes with representative examples to best understand each theme.
Understanding How to Strengthen the Neurology Pipeline With Insights From Undergraduate Neuroscience Students (11) *American Academy of Neurology* Year Published: 2022	Focused on input from undergrad students on their experiences with neuroscience. The survey quantitatively and qualitatively assessed students’ clinical neurology exposure, research experiences, and career goals.	In order to seek how to implement more neuroscience into classrooms based on undergrad student experience with science/neuroscience.	Participants: 1,085 undergraduate students	study was comprised of 1,085 undergraduates in either neuroscience courses or majoring/minoring in neuroscience from across the United States to better understand their clinical neurology experiences and perspectives.	results from an anonymous cross-sectional online survey were studied	Qualitative results include student perspectives from those who did and did not work with a neurologist, describing how they were or were not able to obtain such opportunities. We discuss translating the survey findings into actionable results with opportunities to target the undergraduate neuroscience interest to improve the neurology pipeline
Everyday Neuroscience: A Community Engagement Course (30) *The Journal of Undergraduate Neuroscience Education (JUNE)* Year Published: 2019	service-learning course “Everyday Neuroscience” in which undergraduate students developed 10 neuroscience-relevant for under-resourced high school students; students rand these lab activities with small groups of high-school students	engage interest in neuroscience in the high schoolers; undergraduates increase their awareness of social disparities; improve communication skills in conveying neuroscience principles to a non-specialist audience *	Participants: 43–44 undergraduate students	undergrad students assigned to 1 of 5 team, all teams charged with developing lesson plans for 2 hands-on activities; 10 visits to the high school	undergraduates were asked to write 4 1-page reflections to link community engagement experiences with students’ personal growth + survey at the beginning and end of the about their impressions, what contributed to their learning, what was most interesting and what skills were developed	Compared to the beginning of the course (77%) at the end, 88% of students thought they had something important to contribute to society. For psychological well-being, there was no change in the frequency for students feeling challenged to grow. Attitudes toward teaching and feeling confident to expression their ideas were more positive at the end of the course (77% v 96% *p* < 0.05)
Strategies for the Introduction of Neuroscience for Underrepresented University Students (5) *Journal of College Science Teaching* Year Published: 2011	series of learning experiences during Brain Awareness Week to increase interest in the field of neuroscience	*To encourage URM students to specialize in the neuroscience field by increasing their knowledge of the neuroscience field	Participants: 12,893 registered for BAW, 250 students counted in attendance at academic conferences	Series of four activities – 1. Brain games, 2. Learning activities, 3. Academic conferences, 4. A research poster session during Brain Awareness Week	qualitatively and quantitatively: Likert-scale survey, open-ended questionnaires, Spanish-English bilingual questionnaire	students highly rated presentations on stroke rehabilitation and fMRI in neuroscience, 100% students who responded to open ended questions thought the conference was meaningful, increased knowledge about research techniques
A proposal for undergraduate students’ inclusion in brain awareness week: promoting interest in curricular neuroscience components (31) *The Journal of Undergraduate Neuroscience Education (JUNE)* Year Published: 2014	proposal for the Brain Awareness Week to be a neurophysiology teaching strategy to include undergraduate physiology students in the organization and presentation of BAW events	use Brain Awareness Week (BAW) to increase interest in neuroscience and physiology	Participants: 300 undergraduate and graduate students	BAW lasted for a week	questionnaire given to students to assess if involvement in BAW activities increased their interest in physiology/neuroscience	90% of physiology students said the BAW activities increased their interest in physiology and neuroscience
Introducing high school students to neurophysiology (32) *Advances in Physiology Education* Year Published: 1997	undergrads present physiology research projects to high school seniors	Developed by graduate student volunteers and physiology undergrads. Participants: ~100 high school students each year, unspecified number of undergrads (>100 per year).	Participants: 240 students in their second/third year	Neurophysiology lab, undergrads conduct an experiment over three weeks and present to high school seniors at a 3-h event. All students have the opportunity to both present and hear their classmates present. High schoolers receive a basic pre-lab tutorial to learn basic physiology concept and then ask questions during presentations. The event takes place over four presentation days.	students gave comments on their experiences anecdotally	Quantitative analysis from student comments: high school students claim to become very excited about physiology and leave the campus with a greater feeling of confidence in their ability to succeed in university science. Undergrad presenters feel inspired and appreciate the ability to showcase their work.
Neuroscience Workshops for Fifth-Grade School Children by Undergraduate Students: A University-School Partnership (33) *CBE – Life Sciences Education* Year Published: 2006	University-school partnership: scientist-in-the-classroom visits from undergrads to elementary students on brain structure, disorders, perception. Undergrads develop and implement a fieldwork study.	undergrads learn how to conduct field research using an inquiry-based approach and elementary students learn basic neuroscience concepts	Participants: 99 elementary school students, 8 undergraduate students	As part of a senior seminar course, undergrad psychology students developed a research study to deliver workshops to elementary students and evaluate effectiveness of their teaching. Undergrads created and practiced lessons over the course of 15 weeks, and delivered a single 1-h neuroscience lesson to elementary school students.	pre- and post-test developed by undergrads delivered one week before and after visit testing elementary student’s knowledge of neuroscience concepts. Undergrads evaluated their experience with Likert scale and open-ended questions.	Mixed ANOVA showed workshops improved the children’s knowledge of neuroscience concepts; qualitative analysis of open-ended questions by undergraduates showed they also found their learning experience engaging and productive
Teaching about the brain and reaching the community: undergraduates in the pipeline neuroscience program at the university of Pennsylvania (34) *The Journal of Undergraduate Neuroscience Education (JUNE)* Year Published: 2007	Called the Pipeline Neuroscience Program, this is an educational outreach and mentorship program where undergraduate students teach neuroscience concepts to high school students	This pipeline program mutually serves benefit to the high school students who are being taught neuroscience concepts and career options by undergraduate students, as well as the undergrads who are taught and guided by medical students and neurology residents	Participants: 8 undergraduates, around 26 high school students*	Program was a semester long on a weekly basis, with classes about 90 min long. Upper and lower-level medical students took turns in delivering the lessons, and undergraduates were teaching assistants (TAs) to the high school class and held small group activities.	Students provided informal feedback to their program coordinators; some were selected to take videotaped exit interviews recounting their experiences. Undergraduates were also tested on their knowledge from their seniors from pre- and post-program quizzes. 2007 also introduced a longitudinal evaluation of the students’ college matriculation into neuroscience careers.	Overall feedback from the high schools, undergraduate TAs, medical students, and residents, have been positive with the goals and outcomes of the pipeline program. Many expressed the program increased knowledge of neuroscience, positive impacted on mastery of material, and influence on career goals or plans for a medical subspecialty.
Bridge to neuroscience workshop: An effective educational tool to introduce principles of neuroscience to Hispanics students (35) *PLOS ONE* Year Published: 2019	full-day hands-on workshop in neuroscience education	to identify promising students for the Bridge to the PhD in Neuroscience Program, to increase awareness of neuroscience as a discipline and a career option	Participants: 200 high school students and 424 undergraduate students	*Targeted URM, one-full day workshop, 4 sessions in the workshop	pre and post evaluations, feedback forms	participation in the workshop increases understanding and enthusiasm for neuroscience as a field, high schoolers had more incorrect answers on the pretest than undergraduate students did, mean percentage of correctly answered questions increased for both high school and undergraduates in the posttest
A Capstone Course Where Students Present Contemporary Neuroscience Research to High School Students (36) *The Journal of Undergraduate Neuroscience Education (JUNE)* Year Published: 2020	neuroscience capstone course where students choose and study contemporary neuroscience research articles in depth and then present them to high school students in short videos	For undergrads to apply their neuroscience knowledge and communication skills. High schoolers also benefit from presentations.	Participants: over three years, number of undergrad students varied each year between 10–15. 1 high school class each year.	as part of a semester-long senior course, undergrads developed a research project by reading through scientific literature. Undergrads adapted presentations for their high school audience in the form of posters or videos. Presentation was an hour long.	Undergrads rated the capstone course in a course evaluation quantitatively and qualitatively with open ended questions and 1–10 ratings. High schoolers were not evaluated.	Over 3 years, course ratings averaged 3.87, 4.45, and 4.61 with a positive trend. Anecdotally, undergrads find it a meaningful culminating experience of their undergraduate neuroscience education

## Results

### Publications analysis

The articles were published between 1997 and 2022, with 25/33 (76%) published in the last ten years. Although the articles targeted an undergraduate student population, only about 5/33 (15.2%) explicitly mentioned including or targeting a URM student demographic. Programs targeted at URM students are marked with an asterisk (*) in Tables 2–6. The most common journals for the articles to be published in were the *CBE – Life Sciences Education*, *Journal of Neuroscience Education (JUNE)*, *PLOS One*, *Neurology*, and *Advances in Physiology Education*.

**Table 2:** Case-study/project-based programs.

Title, First Author, Year, Journal	Program type	Program goals	Developers/Participants	Program duration	Program Measures	Program Outcomes
Using case studies as a semester-long tool to teach neuroanatomy and structure–function relationships to undergraduates (14) *The Journal of Undergraduate Neuroscience Education (JUNE)*	Model-based program having students work together with case studies as a tool to teach brain structure–function relationships.	Implement basic and applied neuroanatomy with the fundamentals of structural organization of the nervous system.	Developed by faculty at Denison University Participants: 50 undergraduates in a Biological Psychology course.	Students met three days a week over the 14-week semester and had seven days devoted to presentations and discussions of assigned case studies.	Program was evaluated from quality of student presentations, assessment of knowledge, discussion questions and level of engagement, as well as peer feedback.	85% of students reported positive feedback in using the case studies to learn concepts, as well as more peer engagement. One criticism was feeling more time was needed to learn concepts, feeling some were too “condensed.”
Professor Eric Cannot See: A Project-Based Learning Case for Neurobiology Students (15) *The Journal of Undergraduate Neuroscience Education (JUNE)*	Case study incorporated in an upper-level undergraduate course	Focuses on teaching to critically evaluate primary literature and analyze symptoms and treatment of relevant case-study scenario.	Case study developed by: Eric Ribbensy Participants: N/A	Taught in a Neurobiology of Disease course, case had five scenes and an in-class worksheet, unit was taught in six 75-min class periods.	Tracked progress with in-class and homework assignments, students were asked to thoughtfully identify learning objectives and took a final exam.	Overall class average from unit exam was 83.9%, and some provided positive feedback in wide range of material taught from the case-study.
Using Case Studies to Promote Student Engagement in Primary Literature Data Analysis and Evaluation (16) *The Journal of Undergraduate Neuroscience Education (JUNE)*	Lesson plan for incorporating literature-based neurology case studies into cellular and molecular neuroscience course for upper-level undergraduates.	Increase student engagement with primary/basic neuroscience literature, especially among students interested in clinical healthcare careers	Developed by: faculty in neuroscience program at Carthage Collegey Participants: 29 students	Students worked in small groups to discuss case studies with Pretzel Syndrome and ALS and answered questions; whole class reviewed in larger discussions throughout course.	Assessed learning with take-home exams and gave individual pass/fail grades; qualitative student evaluation of lesson (verbal feedback) and quantitative (Likert scale) before and after lessons.	Students appreciated clinical/real-world applications; increased confidence in understanding neurodevelopment, cellular/molecular neuroscience, and diseases and drugs used in treatments.
The Design, Implementation, and Assessment of an Undergraduate Neurobiology Course using a Project-Based Approach (6) *The Journal of Undergraduate Neuroscience Education (JUNE)*	Project-based approach consisting of student-led work, team-authored research papers, and poster presentations to improve understanding of neuroscience.	Better engage students in upper-level neurobiology course and promote positive attitudes in confidence with material.	Developed by: Professor Zwick at Stockton University Participants: 37 students (31 seniors and 6 juniors).	Students met 2x a week during 15-week semester; each class meeting was 110 min long and taught by 1 instructor. 3 main project themes (neurotoxins, spinal cord injury and learning and memory).	Pre- and post-tests given to students to measure content knowledge, students completed pre- and post-surveys during first and last week of class to measure confidence in neuroscience and attitudes working in team projects.	Greater performance on the post-test compared to the pretest, by end of course students reported significantly higher confidence in neuroscience knowledge.
‘Without A Key’: A Classroom Case Study (17) *The Journal of Undergraduate Neuroscience Education (JUNE)*	Case study using a narrative focused on Locked-in syndrome to engage upper-level undergraduates with neuroanatomy, clinical neuroscience and brain computer technology.	Allow students to work collaboratively to apply their analytical and course-related skills in an open-ended space to extended scientific skill development.	Developed by: faculty in dept. of psychology at Lewis and Clark College Participants: 25 students	Students worked in small groups in upper-level Cognitive Neuroscience course with both lecture and lab components. Case is preceded by brief review lecture and implementation takes 90 min. Class session	students provided anonymous feedback online about the case, 8 feedback questions assess the students’ qualitative experiences rating on a scale based on the goals of the program (specific content questions and general interest)	96% of participants agreed or strongly agree that exercise was “engaging and enjoyable,” 83% agreed it was more interactive than a traditional lectures, and 96% agreed that the case studies increased their knowledge of the topic and syndrome.
Effective Use of Student-Created Case Studies as Assessment in an Undergraduate Neuroscience Course (18) *The Journal of Undergraduate Neuroscience Education (JUNE)*	Students made case-studies used as an assessment tool for an online neurovirology module in a neuroanatomy and physiology course	improving student performance and increasing students’ knowledge and confidence in neuroscience	Developed by: Participants: 24 students	Administered over zoom during weekly 3-h lab period for 4 weeks (week 3–4 used for case study assignment) students used writing practice to write their case studies (graded), students exchanged case studies with their peers and taught/completed case studies with their peers as a low-stakes assessment compared students grades of 1st and final draft of case study.	Students used a Likert scale to self-report their knowledge of content learning objectives and confidence in skills learning objective after completion of the module and the case-study assignment	Grades increased compared from first exam to the final, supporting that interactive writing practices and low-stakes exams can improve performance. Students reported that writing, teaching and completing a neurovirology case-study all increased knowledge – writing ranked higher than teaching or completing.

**Table 3:** Learning tools-based programs.

**Title, First Author, Year, Journal**	**Program type**	**Program goals**	**Developers/ Participants**	**Program duration**	**Program measures**	**Program outcomes**
Learning Neuroscience: An Interactive Case-Based Online Network (ICON) (16) *Journal of Science Education & Technology*	ICON, interactive case-based online network, that is introduced to undergraduate curriculum to bring a different focus on reshaping their thinking in neuroscience	Enhance students' approach to problems, solutions, and insights in neuroscience	Faculty members wrote the cases in the program, expert faculty from FAS, Harvard Medical School, Harvard's teaching hospitals and other academic institution also invited in the development of the program / 12 undergraduates	Applied in Harvard Faculty of Arts and Science curriculum as an honors elective (for 1 semester), students met face to face for 2 hours 1x week in two tutorial groups of 6 students each.	letter grades for the tutorials were assigned according to online participation, contributions to discussions, written case summaries, and final examination - also had students and faculty submit an evaluation of the program as a learning tool	full participation in all modules; thoughtful analysis of the each other's scientific reasoning in the case studies, weekly face to face meetings were lively and conversation picked up from the previous meetings (ICON supported the active participation of the faculty and students' learning)
Online Teaching Tool Simplifies Faculty Use of Multimedia and Improves Student Interest and Knowledge in Science (19) *CBE - Life Sciences Education*	Developed Online Multimedia Teaching Tool (OMTT) in Neuroscience for faculty to use to help deliver online resources in a similar format used in their lectures. Students had unlimited online access for self-exploration on the topic of neuroscience	Developed to help increase student knowledge and interest in neuroscience + simplify instructor adaptation by giving a lecture-based method of digital curriculum	Developed by funding agency, scientists, and programmers. Student participants - 221 Faculty - 5	Supplemented existing curricula in multiple courses, -1st Beta phase released on Parkinson's disease, 2nd phase released OMTT chapters on Ion channels, Synapses. 3rd phase released Functional Neuroanatomy and Biogenic Amines -- developed over 2 years which students and faculty had access to, giving instructional login at the beginning of the semester and results compared at the end of the course	Comparing outcomes for students who volunteered to participate in the program to a control group of students in that class who did not choose to register for OMTT for the course. Conducted survey of user opinion on use of OMTT. Also compared whether students who used OMTT had better performance on traditional in-class forms for assessment.	OMTT used at USC during Fall Semester of 2009 showed registered students did significantly better when instructor used OMTT in lectures and when exam covered material in the OMTT, OMTT in neuroscience viewed positively by students across all 4 courses, also felt instructors' use of OMTT in class helped them understand material better and made them want to self-explore OMTT in their free time
The Lateralizer: A Tool for Students to Explore the Divided Brain (15) *Advances in Physiology Education*	A tool that implements the divided visual field (DVF) technique, introduced as a problem-based learning module in a research methods course, students designed DVF experiments.	To help students explore the differences between the left and right cerebral hemispheres	Participants: 240 upper-level undergraduates, developed by neuroscientists	Program was introduced during a 2-week module in a large research methods course halfway into the semester, large weekly lecture (50 minutes), 2hr laboratory classes a week	Research reports, multiple-choice questionnaires about the lateralization of cognitive function, the anatomy of the brain, and the spatial frequency hypothesis to test student learning outcomes, pretest and posttest.	significant improvement in scores from pretest to posttest, students gained a new knowledge of brain anatomy and connectivity, project added to students’ research skills
The Open PicoAmp: an open-source planar lipid bilayer amplifier for hands-on learning of neuroscience (18) *PLOS ONE*	An open-source lipid bilayer amplifier was developed for use in neuroscience introductory courses.	Hands-on approach to learning neuroscience to teach about different electrical properties of the cell membrane.	Sessions coordinated by adjuncts instructors and older students that were trained. Study approved by Institutional Ethical Committee of the School of Medicine of the Université Libre de Bruxelles	It was about a 4-hour laboratory session. Usually, one group of two students is assigned per bilayer setup. To allow the session to run smoothly and maximize the interactions with the students, one instructor for two groups of students is an optimal configuration. Lab sessions at our institution are run with ten bilayer setups working simultaneously.	Program was studied by the adjuncts instructors and older students which have been previously trained.	In a typical four hours session, all students were able to obtain the lipid bilayer formation and study its electrical properties. Also, a majority of attendees could actually succeed to observe the single channel currents. This was a good performance, as it is a rather delicate experiment and for many students this was their first experience in such a laboratory environment.
Neurogaming Technology Meets Neuroscience Education: A Cost-Effective, Scalable, and Highly Portable Undergraduate Teaching Laboratory for Neuroscience (12) *The Journal of Undergraduate Neuroscience Education (JUNE)*	A teaching lab with equipment to incorporate into an introductory neuroscience curriculum. The devices used to EEG machines for the students to use as a research tool and enhance student's' introduction into neuroscience	Use cost-effective tools and implement a teaching lab to be able to expose students to laboratory/research experience	Participants: 268 undergraduates, but only 150 students fully completed the evaluation survey	EEG headsets and software were incorporated to students' lab sessions over the course of their semester, with each lab session about 1-hour long	Students took in-class surveys with a 5-point Likert scale with responses consolidated into three findings: enjoyment, usefulness, and improvement on understanding; and their completed labs were looked into	Students have shown that the EEG-incorporated labs were enjoyable and increased their knowledge in neuroscience concepts and EEG, as well as provided them firsthand research experience.
Using A Contrast Illusion to Teach Principles of Neural Processing (13) *The Journal of Undergraduate Neuroscience Education (JUNE)*	an interactive laboratory exercise to challenge undergrads to use observational measurements of a visual contrast illusion to study neural activity	to understand the effectiveness of this active learning exercise in increasing student understanding of how perception is shaped by neural circuits in the retina	Developed by neuroscientists. Participants: 112 students.	Students conducted simple psychophysical experiments to measure thresholds for detecting illusory spots under various conditions and described their results in a laboratory assignment.	in a pre and post survey, students’ confidence and practical understanding of 3 main concepts neural processing were evaluated on a Likert Scale.	students had significant improvements in content knowledge and increased confidence in ability to demonstrate understanding after the lab. students had overall positive attitudes towards the lab.
Hands-on Undergraduate Experiences Using Low-Cost Electroencephalography (EEG) Devices (17) *The Journal of Undergraduate Neuroscience Education (JUNE)*	Implementing EEG through MUSE headbands.	To determine whether or not EEG can help students learn better about their brain	Participants: 13 juniors and 13 seniors.	Students utilized EEG in classrooms to see how the brain functioned while doing several different brain exercises. This consisted of trying new tastes and answering different questions.	Students reflected on their own EEG data and filled out surveys to determine whether or not the implementation of EEG had an effect on understanding the brain.	Students felt more motivated to work on their current tasks and projects due to the EEG. Many students expressed enthusiasm and excitement for learning more.
Signal: A Neurotransmission Board Game (14) *The Journal of Undergraduate Neuroscience Education (JUNE)*	use of a board game designed to test students' understanding of core concepts of core concepts covered in introductory neuroscience courses - action potentials and synaptic transmission	game-based learning serves an effective and engaging tool to review fundamentals of neurotransmission in science courses - allowed students to develop skills and gained a greater understanding of neuroscience	Angel W. Kaur/ 36 students (38 played the game but 2 opted out)	participants were undergraduate students enrolled in "Fundamentals of Neuroscience" course; students played signal during week 13 of semester an in-class activity in groups 4-5 players - carried out in 2 sections of course in 1 semester	After completing the board game, students asked to complete survey on their impressions of the exercise (rated their agreement to statements)	80.5% (n=24) agreed or strongly agree (n=5) that the game helped them assess their knowledge and understanding of action potential and 83.3% (n=22) + (n=8) agreed that it helped their understanding of synaptic transmission. 30 students agreed that it allowed to develop communication skills as they talked to their peers about scientific concepts

**Table 4:** Multidisciplinary undergraduate courses.

Title, First Author, Year, Journal	Program type	Program goals	Developers/Participants	Program duration	Program measures	Program outcomes
Neuroscience and Sustainability: An Online Module on “Environmental Neuroscience” (8) *The Journal of Undergraduate Neuroscience Education (JUNE)*	Neuroscience concepts were incorporated into a mid-level biopsychology course through an online “Environmental Neuroscience” module	To enhance students’ understanding of neuroscience and how it can contribute to both sustainability efforts and other scientific disciplines (e.g.: environmental psych)	Created by university faculty in Dept. of Psychology, Program of Neuroscience at U St. Thomas. Participants: 26 students	The module was conducted over the course of the semester, with an “Environmental Stewardship Portfolio” due at the end of the term. Students wrote reflection papers based on five articles of their choosing, each due every 2–3 weeks. Most of the work was done outside of class, meaning the module did not take up class time.	Students’ papers were given critiques for revisions, completed an anonymous evaluation of the module, and took a knowledge test with results compared to a control group (n = 27) of students who did not take the online module but the same course.	Students mostly showed significant increase in knowledge and awareness of neuroscience and how it connects to other disciplines of science, like environmental issues, and also scored higher than the control group on the knowledge assessment (median score of 67.8, SD of 16.9).
Integrating Brain Science into Health Studies: An Interdisciplinary Course in Contemplative Neuroscience and Yoga (9) *The Journal of Undergraduate Neuroscience Education (JUNE)*	Undergraduate health class combining contemplative neuroscience with yoga practice to introduce students who might otherwise avoid taking explicit neuroscience classes to elements of neuroscience in an integrative and accessible manner	Engage a broader student population in neuroscience learning, beyond those already interested in science, by relating neuroscience to health concepts through the interdisciplinary field of contemplative neuroscience	Developed by faculty/researchers at University of St. Thomas in MN; 80 students participated between fall 2012 and spring 2015 in sections of 10–16 students	Semester-long course that met for 1 h and 45 min once a week with ~50–55 min of lecture/discussion followed by yoga practice relevant to the contemplative neuroscience topics previously discussed; required readings were accessible to a lay audience; students were required to complete a weekly journal and one final exam	One cohort of 12 students was administered a 10-item multiple choice neuroscience pre/posttest; at the end of the semester, students complete a course rating assessing their progress in course objectives on a Likert scale	Significant increase in scores on neuroscience knowledge posttest; average student course ratings indicated that students had more positive feelings about the field of neuroscience after taking the course
Reading the Brain: An Interdisciplinary First-Year Seminar on the Intersection of Neuroscience, Literature, and Popular Culture (10) *The Journal of Undergraduate Neuroscience Education (JUNE)*	interdisciplinary 1st-year seminar that is taught on the intersection of neuroscience, literature, films, plays and advertisement + ways that brain science can help people understand why and how we read taught by cognitive neuroscientist and literary scholar	to highlight benefit of using non-technical sources to introduce students to scientific concepts and value of teaching collaboratively across disciplinary boundaries. Engaging students in cross-disciplinary thinking and helping neuroscience students grasp difficult topics (achieve better learning outcomes)	Developed by neuroscience instructors. Participants: 32 students (for 2 years)	course was a part of first-year seminar program that is limited to small # of students. Course was taught in 2 subsequent academic years. Class met weekly for two 75-min periods. Used discussion format course was divided into 8 segments	- assessed the degree to which the student’s writing has improved over the semester (compared results on students’ 1st analytical paper to performance on students’ 2nd analytical paper), - assessed analytical paper over 3 metrics: literary analysis, brain literacy and interdisciplinary to see impact - administered mid-semester and end-of semester evaluation to assess degree of which the course was successfully implemented according to students	students had positive view on course content and course interdisciplinary writing improved over the course and ability to make interdisciplinary connections in both class and written assignments. 68% (11) of students went on to declare majors, 11% of students declared minors in STEM disciplines
Drugs, the brain, and behavior: a graduate student-run comprehensive course in neuroscience (12) *The Journal of Undergraduate Neuroscience Education (JUNE)*	2 semester upper-level course that employs an interdisciplinary approach to normal and altered nervous system function; aimed toward both undergraduates and PhD students	expose students to neuroscience + knowledge in neurological and neuropsychiatric illness; promote scientific literacy in undergraduates and provide pedagogical experience for PhD students	Participants: 240 students in their second/third year, developed by neuroscientists	run and taught by Ph.D. students in the Interdisciplinary program in Neuroscience at Georgetown University/24 students	students enrolled in fall semester filled out a questionnaire after the midterm (agreements with 9 statements of proficiency in topics taught) – asked to estimate agreement prior to start of the course and at the time of the midterm compared final and midterm exam scores	in the fall semester, students showed significant improvement (21%) in raw scores between the midterm and final examinations, all students rated themselves at a higher level in their proficiency of the topics taught compared to the beginning of the course
Social neuroscience at the college of saint rose: the art of team teaching in emerging areas of psychological science (13) *The Journal of Undergraduate Neuroscience Education (JUNE)*	a new undergraduate level course for neuroscience that merges psychology and neuroscience	to develop an effective team-taught course in social neuroscience at the undergraduate level, provide undergraduate students with access to new, developing content merging social psychology and neuroscience	Participants: 15 students, neuroscience faculty at The College of Saint Rose developed the program	traditional lecture-style course with seminar-style course combination, students were assigned readings from text and one student was deemed discussion leader for that text, three exams delivered throughout the term, students required to develop research proposal and present it to the class	students were given a anonymous questionnaire and demographic survey at the end of the course, two-tailed one sample t-tests used to analyze data	students reported gaining a better understanding of the material through team teaching vs. single teacher teaching, students reported enjoying taking the newly designed course

**Table 5:** Research opportunity programs.

Title, First Author, Year, Journal	Program type	Program goals	Developers/Participants	Program duration	Program measures	Program outcomes
Evaluation of an undergraduate neuroscience research program at the University of Kentucky (7) *The Journal of Undergraduate Neuroscience Education (JUNE)*	neuroscience summer research experience	*Expose undergraduate students to various graduate careers, inform them of neuroscience opportunities, increase URM students, give them research project of their own	Members at the University of Kentucky, department of Anatomy and Neurobiology, Participants: 10 selected from applicant pool every - which is about 50 students, overall, 80 students	throughout the summer in 5 different components - 1. neuroscience course, 2. independent research projects, 3. lunchtime seminars, 4. summer symposium, 5. oral presentations at the end of the summer	annual end of summer survey	participation in this program may have strengthened students’ applications to graduate schools, high satisfaction with the material students were exposed to, successfully increased URM in neuroscience research, participation in program increases chances students will apply to graduate school
Partnerships in Neuroscience Research Between Small Colleges and Large Institutions: A Case Study (4) *The Journal of Undergraduate Neuroscience Education (JUNE)*	Neuroscience internship program for students (ideally 2nd or 3rd years) at Stonehill College to participate in either the Basic Research or Clinical Research division of the Laboratory of Neuroscience in the Department of Psychiatry, at Harvard Medical School	Goal is to provide students in smaller colleges a program within a larger institution with resources to enrich their curricula and extracurricular experience; helps to give students an experience they can utilize when applying to graduate programs	Participants: 35 total interns have completed this program and went onto graduate programs; 25 responded to a survey done by the authors	An intern would then work 8–12 h/week during the school year, but then increase their workload to full time for 10 weeks (35 h/week) during the summer typically through the Stonehill Undergraduate Research Experience	Program studied based on student performance and completion of the program. Survey was done comparing the interns’ initial goals once leaving the program versus actual outcomes on where they are after college. Also largely studied on student feedback and impressions	Many stayed consistent with where they wanted to go versus where they ended up, specifically with doctoral programs (i.e.: medical school) with ~13. Many more students actually went into research with masters or PhD, with not many having that as an initial goal (2–3 responded as a goal, 8–10 responded as an outcome)
Integrating Research into the Undergraduate Curriculum: 1. Early Research Experiences and Training (27) *The Journal of Undergraduate Neuroscience Education (JUNE)*	This article expands upon the information presented in workshop discussions, focusing on ways to promote early research opportunities.	To figure out different benefits and challenges of implementing research in the undergrad experience	N/A	Studied different articles and information from workshops to sort out details of what is beneficial or not for students.	Program was studied based on how students from different backgrounds (experienced or non-experienced) were able to perform in their research.	Found that it is beneficial to support students as they discover science, grow in confidence and competence, and move toward a future that will be well served by a diverse group of thoughtful, curious, well-prepared individuals as they are able to enter the workforce.
Integrating Research into the Undergraduate Curriculum: 3. Research Training in the Upper-level Neuroscience Curriculum (28) *The Journal of Undergraduate Neuroscience Education (JUNE)*	This article expands upon the information presented in workshop discussions, focusing on ways to promote early research opportunities.	To figure out different benefits and challenges of implementing research in the undergrad experience	N/A	Studied different articles and information from workshops to sort out details of what is beneficial or not for students.	Program was studied based on how students from different backgrounds (experienced or non-experienced) were able to perform in their research.	Found that it is beneficial to support students as they discover science, grow in confidence and competence, and move toward a future that will be well served by a diverse group of thoughtful, curious, well-prepared individuals as they are able to enter the workforce.
Building the Neurology Pipeline with Undergraduate Students in Research and Clinical Practice (29) *American Academy of Neurology*	Article studies different logistics, potential benefits and challenges on implementing undergrad neuroscience clinical research.	This article discusses how working with undergraduate students represents a unique opportunity to expose students to neurology through research and clinical care while also generating many beneficial experiences for the student and the neurologist. We outline potential benefits, and logistical considerations, involved in working with undergraduates.	Program was developed by Dr. Minen, and a group of undergraduate students that were able to access through different articles in order to accumulate enough data to reason that clinical neurology is important.	Various different articles and data collected from previous research was studied to determine the different implications of clinical neuroscience.	Various different articles and data collected from previous research was studied to determine the different implications of clinical neuroscience.	We incorporated undergraduate student perspectives regarding the impact of their experiences on their personal and professional lives. In total, we received reflections (*n* = 32), emails (*n* = 4), applications for opportunities (*n* = 7), and a program feedback survey (*n* = 1). In a process considered exempt by the Wake Forest Baptist Institutional Review Board, we qualitatively analyzed these data into themes with representative examples to best understand each theme.
Understanding How to Strengthen the Neurology Pipeline With Insights From Undergraduate Neuroscience Students (11) *American Academy of Neurology*	Focused on input from undergrad students on their experiences with neuroscience. The survey quantitatively and qualitatively assessed students’ clinical neurology exposure, research experiences, and career goals.	In order to seek how to implement more neuroscience into classrooms based on undergrad student experience with science/neuroscience.	Program was developed by Dr. Minen and undergrad students to help improve pipeline.	study was comprised of 1,085 undergraduates in either neuroscience courses or majoring/minoring in neuroscience from across the United States to better understand their clinical neurology experiences and perspectives.	results from an anonymous cross-sectional online survey were studied	Qualitative results include student perspectives from those who did and did not work with a neurologist, describing how they were or were not able to obtain such opportunities. We discuss translating the survey findings into actionable results with opportunities to target the undergraduate neuroscience interest to improve the neurology pipeline

**Table 6:** Extracurricular Programs.

Title, First Author, Year, Journal	Program type	Program Goals	Developers/Participants	Program duration	Program measures	Program outcomes
Everyday Neuroscience: A Community Engagement Course (30), *The Journal of Undergraduate Neuroscience Education (JUNE)*	service-learning course “Everyday Neuroscience” in which undergraduate students developed 10 neuroscience-relevant for under-resourced high school students; students rand these lab activities with small groups of high-school students	engage interest in neuroscience in the high schoolers; undergraduates increase their awareness of social disparities; improve communication skills in conveying neuroscience principles to a non-specialist audience *	collaboration with faculty of university + teacher and admin of nearby high school/43–44 undergraduate students	undergrad students assigned to 1 of 5 team, all teams charged with developing lesson plans for 2 hands-on activities; 10 visits to the high school	undergraduates were asked to write 4 1-page reflections to link community engagement experiences with students’ personal growth + survey at the beginning and end of the about their impressions, what contributed to their learning, what was most interesting and what skills were developed	Compared to the beginning of the course (77%) at the end, 88% of students thought they had something important to contribute to society. For psychological well-being, there was no change in the frequency for students feeling challenged to grow. Attitudes toward teaching and feeling confident to expression their ideas were more positive at the end of the course (77% v 96% *p* < 0.05)
Strategies for the Introduction of Neuroscience for Underrepresented University Students (5) *Journal of College Science Teaching*	series of learning experiences during Brain Awareness Week to increase interest in the field of neuroscience	*To encourage URM students to specialize in the neuroscience field by increasing their knowledge of the neuroscience field	Participants: 12,893 registered for BAW, 250 students counted in attendance at academic conferences, developed by members at The University of Puerto Rico (URM)	Series of four activities – 1. Brain games, 2. Learning activities, 3. Academic conferences, 4. A research poster session during Brain Awareness Week	qualitatively and quantitatively: Likert-scale survey, open-ended questionnaires, Spanish-English bilingual questionnaire	students highly rated presentations on stroke rehabilitation and fMRI in neuroscience, 100% students who responded to open ended questions thought the conference was meaningful, increased knowledge about research techniques
A proposal for undergraduate students’ inclusion in brain awareness week: promoting interest in curricular neuroscience components (31) *The Journal of Undergraduate Neuroscience Education (JUNE)*	proposal for the Brain Awareness Week to be a neurophysiology teaching strategy to include undergraduate physiology students in the organization and presentation of BAW events	use Brain Awareness Week (BAW) to increase interest in neuroscience and physiology	Participants: 300 undergraduate and graduate students, physiology focused researchers developed the proposal	BAW lasted for a week	questionnaire given to students to assess if involvement in BAW activities increased their interest in physiology/neuroscience	90% of physiology students said the BAW activities increased their interest in physiology and neuroscience
Introducing high school students to neurophysiology (32) *Advances in Physiology Education*	undergrads present physiology research projects to high school seniors	Developed by graduate student volunteers and physiology undergrads. Participants: ~100 high school students each year, unspecified number of undergrads (>100 per year).	Participants: 240 students in their second/third year, developed by neuroscientists	Neurophysiology lab, undergrads conduct an experiment over three weeks and present to high school seniors at a 3-h event. All students have the opportunity to both present and hear their classmates present. High schoolers receive a basic pre-lab tutorial to learn basic physiology concept and then ask questions during presentations. The event takes place over four presentation days.	students gave comments on their experiences anecdotally	Quantitative analysis from student comments: high school students claim to become very excited about physiology and leave the campus with a greater feeling of confidence in their ability to succeed in university science. Undergrad presenters feel inspired and appreciate the ability to showcase their work.
Neuroscience Workshops for Fifth-Grade School Children by Undergraduate Students: A University-School Partnership (33) *CBE - Life Sciences Education*	University-school partnership: scientist-in-the-classroom visits from undergrads to elementary students on brain structure, disorders, perception. Undergrads develop and implement a fieldwork study.	undergrads learn how to conduct field research using an inquiry-based approach and elementary students learn basic neuroscience concepts	Developed by undergrads. Participants: 99 elementary school students, 8 undergraduate students	As part of a senior seminar course, undergrad psychology students developed a research study to deliver workshops to elementary students and evaluate effectiveness of their teaching. Undergrads created and practiced lessons over the course of 15 weeks, and delivered a single 1-h neuroscience lesson to elementary school students.	pre- and post-test developed by undergrads delivered one week before and after visit testing elementary student’s knowledge of neuroscience concepts. Undergrads evaluated their experience with Likert scale and open-ended questions.	Mixed ANOVA showed workshops improved the children’s knowledge of neuroscience concepts; qualitative analysis of open-ended questions by undergraduates showed they also found their learning experience engaging and productive
Teaching about the brain and reaching the community: undergraduates in the pipeline neuroscience program at the university of Pennsylvania (34) *The Journal of Undergraduate Neuroscience Education (JUNE)*	Called the Pipeline Neuroscience Program, this is an educational outreach and mentorship program where undergraduate students teach neuroscience concepts to high school students	This pipeline program mutually serves benefit to the high school students who are being taught neuroscience concepts and career options by undergraduate students, as well as the undergrads who are taught and guided by medical students and neurology residents	Developed at University of Pennsylvania School of Medicine; participants: 8 undergraduates, around 26 high school students*	Program was a semester long on a weekly basis, with classes about 90 min long. Upper and lower-level medical students took turns in delivering the lessons, and undergraduates were teaching assistants (TAs) to the high school class and held small group activities.	Students provided informal feedback to their program coordinators; some were selected to take videotaped exit interviews recounting their experiences. Undergraduates were also tested on their knowledge from their seniors from pre- and post-program quizzes. 2007 also introduced a longitudinal evaluation of the students’ college matriculation into neuroscience careers.	Overall feedback from the high schools, undergraduate TAs, medical students, and residents, have been positive with the goals and outcomes of the pipeline program. Many expressed the program increased knowledge of neuroscience, positive impacted on mastery of material, and influence on career goals or plans for a medical subspecialty.
Bridge to neuroscience workshop: An effective educational tool to introduce principles of neuroscience to Hispanics students (35) *PLOS ONE*	full-day hands-on workshop in neuroscience education	to identify promising students for the Bridge to the PhD in Neuroscience Program, to increase awareness of neuroscience as a discipline and a career option	Participants: 200 high school students and 424 undergraduate students overall, each session has about 30 students, developed by 9 graduate students at Michigan State University	*Targeted URM, one-full day workshop, 4 sessions in the workshop	pre and post evaluations, feedback forms	participation in the workshop increases understanding and enthusiasm for neuroscience as a field, high schoolers had more incorrect answers on the pretest than undergraduate students did, mean percentage of correctly answered questions increased for both high school and undergraduates in the posttest
A Capstone Course Where Students Present Contemporary Neuroscience Research to High School Students (36) *The Journal of Undergraduate Neuroscience Education (JUNE)*	neuroscience capstone course where students choose and study contemporary neuroscience research articles in depth and then present them to high school students in short videos	For undergrads to apply their neuroscience knowledge and communication skills. High schoolers also benefit from presentations.	Program course developed by neuroscientist/professor; presentations developed by undergrads. Participants: over three years, number of undergrad students varied each year between 10–15. 1 high school class each year.	as part of a semester-long senior course, undergrads developed a research project by reading through scientific literature. Undergrads adapted presentations for their high school audience in the form of posters or videos. Presentation was an hour long.	Undergrads rated the capstone course in a course evaluation quantitatively and qualitatively with open ended questions and 1–10 ratings. High schoolers were not evaluated.	Over 3 years, course ratings averaged 3.87, 4.45, and 4.61 with a positive trend. Anecdotally, undergrads find it a meaningful culminating experience of their undergraduate neuroscience education

### Programmatic content analysis

To better assess the different measures of success with each pipeline program, the 33 programs were classified into subtypes of pipeline programs implemented for undergraduate students.

Six programs (6, 14–18) emphasized student involvement in case-study-based or collaborative-based projects (Table 2). Case-study-centered programs required students to analyze literature-based case studies on neurological illnesses. In project-based approaches, students presented their findings on neurological themes to peers, fostering teamwork and enhancing scientific literature analysis skills (16). Students provided feedback and assessed their knowledge through pre- and post-assessments and surveys.

Eight programs (19–26) involved learning tools and activities to help enrich the undergraduate classroom (Table 3). Interactive modules augmented students’ current curriculum, introducing them to various neurological conditions and procedures, from Parkinson’s disease to electroencephalograms (EEG). Progress was recorded frequently, and participation, engagement, knowledge reports, and final exams determined grades. Both students and faculty evaluated overall effectiveness.

Five programs (8–10, 12, 13) were integrative courses, with neuroscience and other science and non-science-related subjects for a unique perspective on neurological concepts (Table 4). These interdisciplinary disciplines ranged from media to art to convey neurological concepts by catering to students’ diversified interests through cross-disciplinary learning. One program, for example, taught students’ neural mechanisms through yoga and other mindful habits (9). Other courses focused on broader methodological analyses, such as the intersection between neuroscience with technology or environmental issues. Toward the end of these programs, data for student progress was collected through open-ended questionnaires, writing reflections, or final examination scores.

Six programs (4, 7, 11, 27–29) were internships or REU (Research Experience for Undergraduates) programs, exposing students to firsthand clinical or field research opportunities to pique their interests in neurology (Table 5). Students were able to attain valuable technical skills, which they could utilize in future professional endeavors. Furthermore, students were informed of different career paths with which they could specialize in neurology, which were not just healthcare-driven but also research-driven. One program, for instance, introduced students to experiences that can reinforce their confidence in research, such as attending weekly seminars and presenting their research in symposia at the end of the program (7). These programs were often implemented over the summer, allowing students to better immerse themselves in the experience compared to other short-term programs. Completion rates, qualitative feedback, and surveys measured program outcomes. Longitudinal assessments were conducted by some programs to gage how students augmented their experiences by measuring career progression into graduate studies. In one program, many students who originally expressed an interest in matriculating into medical school remained interested in this pursuit. In contrast, others who did not share this initial interest ended up enrolling in graduate programs, such as master’s or doctoral programs (4). Programs also assessed student feedback and impressions to assess how well they enriched students’ experiences.

Eight programs (5, 30–36) were extracurricular workshops or capstone projects for students to participate in and further their neuroscience knowledge. Some of these programs also involved the direction of medical students or neurology residents, and undergraduates presented their applied knowledge to a younger audience, from K-12 students (Table 6). Through interactive field studies and presentations, undergraduates taught core concepts, such as brain structure, disorders, and physiology, in a comprehensible manner to children and adolescents. These programs benefited both high school students and undergraduates, with the latter gaining mentorship and encouragement by medical students and residents. Feedback was largely positive, with undergraduates expressing gained confidence in their ability to educate a younger audience on complex topics. Pre- and post-assessments were given to the younger students to compare their learning outcomes, and surveys were given to both undergraduates and their students to assess their experiences in this type of environment. Other programs had undergraduates participating in learning activities, such as Brain Games or attending academic conferences to reaffirm their interest in learning newfound neurology-related concepts (31).

### Programmatic assessment

In the case-study or project-based programs, one program (14) utilized qualitative methods of measurement, largely analyzing students’ feedback after completing the program. While the remaining five programs (6, 15–18) utilized a mixed methods analysis of the programs. Qualitative data came from student feedback, with many expressing increased knowledge of neurological concepts and lasting positive impressions. Many students appreciated the case studies, as they allowed a stronger grasp of learning objectives and application of their knowledge to real-life scenarios. Quantitative data came from surveys rating students’ experiences (usually on a Likert scale), comparison of grades on pre-and post-assessments, and completion of assignments. All six programs concluded that students felt more confident in their abilities to understand neurological concepts.

Of the eight pipeline programs (19–26) with interactive learning tools in neuroscience courses or labs, only two (19, 22) utilized qualitative data from student feedback and overall completion of the modules or activities to measure program outcomes. Four programs (21, 24–26) were assessed with quantitative methods, evaluating students’ recorded answers from pre- and post-surveys, questionnaires, and exams. Two programs (20, 23) used a mixed methods approach, with qualitative data from student feedback and quantitative data from Likert-scale ratings, completed lab reports, and a comparison of course performance with a control student group. All programs reported students having increased content knowledge and confidence in conveying this comprehension.

Of the six pipeline programs (4, 7, 11, 27–29) designed to expose undergraduate students to extracurricular research experience, four programs (11, 27–29) were assessed using qualitative methods, largely from student performance in the labs and feedback from working in a lab. Two programs (4, 7) were measured with mixed methods, assessing qualitative data from student impressions and feedback and quantitative data from survey responses. All programs concluded that participation in labs left students with positive impressions and enriched their experiences by gaining research exposure.

In the five pipeline programs (8–10, 12, 13) that involved interdisciplinary neuroscience courses, three programs (9, 12, 13) were measured with only quantitative methods, assessing students’ pre- and post-exam scores and numerical ratings from surveys or questionnaires. Two programs (8, 10) were measured with a mixed methods approach, with quantitative data from examination scores after course completion or numerical ratings from questionnaires and qualitative data from students’ quality of portfolio work from the courses, written reflections, or verbal feedback and attitudes.

In the eight capstone-based programs (5, 30–36), six of these programs (5, 30, 33–36) were measured using a mixed methods approach, with quantitative data from numerical surveys, statistical analyses, or pre-and post-exam score comparisons, and qualitative data from students’ verbal feedback or open-ended questionnaires. Two programs (31, 32) were measured with solely qualitative data, using anecdotal evidence or open-ended questionnaires.

## Discussion

Our systematic review introduced a diverse range of pipeline programs and experiences that can inspire undergraduate students to pursue a career in neuroscience or neurology. Ranging from collaborative projects to interdisciplinary approaches, all programs are intended to deliver neuroscience concepts and potential careers. Programs that exhibited the greatest support in facilitating undergraduates’ interest in neuroscience prioritized partnerships with other student populations and offered primary research experience.

Uniquely, involving undergraduates in teaching younger students proved to be valuable. Undergraduates could improve their presentation and communication skills by teaching younger children about neuroscience, and the younger students gain exposure to new concepts in neurology that may inspire them to explore the field in the future. Capstone projects and research opportunities allowed students to immerse themselves in first-hand research and develop their own curriculum, leaving them with a firmer grasp of learned skills and experiences they can build upon.

The continuation of these programs can enhance students’ knowledge of neurology and career opportunities within the field. The integration of other disciplines was a unique aspect of neurology pipeline programs, which attracted students and enabled interactive learning. By incorporating these methods into existing curricula, we can cultivate a diversified and knowledgeable group of students to pursue neurology-related specializations. It can also broaden opportunities for students who are uncertain about their career paths or are underrepresented in these areas of study.

Another relevant consideration from this systematic review is the effects of the COVID-19 pandemic on attracting undergraduates into the field of neurology, as well as the delivery and availability of pipeline programs. As a result of the pandemic, undergraduates may feel anxious or uncertain about future careers, employment prospects, and continuing education. In a recent survey regarding the future of neurological practice post-pandemic, neurology residents expressed concern about the significant proportion of patients forced to postpone appointments, a lack of training in emergency response, and the need to adapt to teleneurology (4). Undergraduates already involved in patient care or neurology research during the pandemic may develop similar concerns about pursuing a neurology career. At the same time, those just learning about the field may be discouraged from getting involved with pipeline programs. Furthermore, the pandemic has had an impact on the delivery and availability of neurology pipeline programs. A systemic review of neurology training programs during the pandemic indicated a change in clinical routine for neurology residents, reduced research activities, and delivery of education via online services rather than in-person (11). Yet, many neurology residents reported having sufficient facilities to continue neurology research remotely. This offers a mixed outlook on pipeline programs during and post-pandemic. On the one hand, there may be limited access to in-person and clinical activities for undergraduate students. On the other hand, they may have increased access to virtual opportunities for research that may not have been available before. Even if virtual neurology pipeline programs are more available, that does not mean that all undergraduates will have equal financial opportunity or time to participate (11).

### Limitations

The screening process of this systematic review has resulted in the exclusion of multiple articles that presented promising pipeline programs designed for an undergraduate audience due to a lack of implementation or no measurable/irrelevant outcomes. Of those implemented, some programs were excluded from this systematic review as they were not designed to inspire students to explore neurology but to teach general skills, such as reading scientific journals, enacting collaboration, or simply passing a class.

Of the programs studied, a significant limitation was the extent to which undergraduate encouragement into neurology was achieved. Most programs were short-term; therefore, no insight was provided after program completion into how these students went on to strengthen their interests. Longitudinal investigations could provide deeper insight into whether these programs do play a transformative role in students’ career preparation or decision to pursue neurology. However, this raises significant challenges due to numerous reasons. In the time between students’ participation in an early-exposure pipeline program and their ultimate career choice, they may undergo various educational and/or personal experiences that may ultimately shape their decision, whether in or out of neurology, making it harder to gage the success of a particular program. Data collection on the long-term career trajectories of program participants can also be logistically challenging. It often requires sustained tracking efforts, which may not be feasible for all pipeline programs, especially those with limited resources. Also, due to the duration these programs can entail, students may face attrition in wanting to continue participating beyond a program’s formal conclusion.

Another drawback to these programs is the geographical accessibility to certain neurology pipeline programs. Limited access to neurology pipeline programs may be especially prevalent in more rural or underserved areas, of which underrepresented students may reside as well, ultimately impacting program representation and participation. Additionally, some programs may be limited in their funding or resources available to extend to a broader student population and thus, provide them with enriching opportunities. These caveats in geographical and resource inaccessibility should be addressed toward efforts in the improvement of neurology pipeline programs and their overall effectiveness.

### Future directions

Future research is necessary to investigate interactions between mixed student populations and how this can help further deepen undergraduates’ interests in neurology. While this review discussed programs including students outside of undergraduates, they highlighted the benefit in fostering engagement and encouragement to undergraduates. Collaboration between trainees at different educational levels can provide a better scope on the effects of the neurology pipeline by facilitating undergraduate student interest in neurology, and thus provide a wider view of programs’ benefits. Emphasizing the importance of mentorship and networking between undergraduate trainees and physicians or graduate students can be integral in facilitating students’ interest and pursuit of neurology. Programs should work to actively connect students to neurology professionals and establish these bonds so that they can help guide students throughout their academic and professional careers (5). Current strategies in tracking the efficacy of neurology pipeline programs, from progress tracking, student feedback collection, and long-term impact assessments, can also shed insight into how students feel more assured in their academic and career choices if augmented with this developed connection and resource of a neurology professional with which they can turn to for guidance. Overall, these programs can be expanded to include programs specifically designed for undergraduates’ professional readiness in neurology, with mentorship and personal connections sustaining their interest and building relevant knowledge and skills for the workforce.

More importantly, future programs should target the inclusion of underrepresented minorities, as only 15% of programs in this review did so. Diversification within neurology not only introduces unique perspectives and problem-solving skills but also improves the workforce of practicing neurologists and promotes broader undergraduate interest, diminishing the gap within the workforce (6). To improve in fostering diversity and inclusion of underrepresented undergraduate students in neurology pipeline programs, programs should actively prioritize the recruitment of students from underrepresented backgrounds and advocate for the support and guidance of these students into neurology-related careers, thus contributing to a more inclusive and representative workforce, with people bringing to the table a wide array of expertise and unique talents that benefits the field as a whole.

To address the challenges posed by geographical and funding or institutional limitations, we must consider innovative approaches to improving neurology pipeline programs. As shown through some program outlines in this study, designing interdisciplinary programs that integrate neurology with other fields of student interests, such as psychology, art, or technology, can provide students with a more comprehensive view of neurology and its applications, but more importantly, it allows smaller institutions to implement programs still relevant to neurology, and utilize available resources to garner a broader student population (9). These institutions can also conduct collaborative efforts with local community colleges or high schools in designing pipeline programs that not only sustain undergraduates’ interests in neurology but also identify and encourage younger students’ excitement about neurology, broadening the reach of these programs and diminishing geographical disparities. Furthermore, embracing a hybrid or remote format for existing or prospective programs, as we have seen from the COVID-19 pandemic, also improves accessibility for students who may be unable to attend in person and even participate in programs that may not be offered at their institution (4). In closing, the need to improve undergraduate-targeted neurology pipeline programs lies not only in the recognition and mitigation of these discussed limitations but also in the pivotal role these programs can play in shaping a dynamic and equitable future for the field of neurology, driven by the talent and diversity of the next generation of neurologists.

## Data availability statement

The original contributions presented in the study are included in the article/supplementary materials, further inquiries can be directed to the corresponding author.

## Author contributions

MM: Conceptualization, Data curation, Formal analysis, Investigation, Methodology, Project administration, Resources, Supervision, Validation, Writing – original draft, Writing – review & editing. RA: Conceptualization, Data curation, Formal analysis, Investigation, Methodology, Writing – original draft, Writing – review & editing. IY: Conceptualization, Data curation, Formal analysis, Investigation, Methodology, Writing – original draft, Writing – review & editing. KO: Conceptualization, Data curation, Formal analysis, Investigation, Methodology, Writing – original draft, Writing – review & editing. JE: Data curation, Formal analysis, Investigation, Methodology, Writing – original draft, Writing – review & editing. AK: Data curation, Formal analysis, Investigation, Writing – original draft, Writing – review & editing. NL: Data curation, Formal analysis, Investigation, Writing – original draft, Writing – review & editing. CP: Conceptualization, Data curation, Formal analysis, Investigation, Methodology, Project administration, Resources, Software, Supervision, Validation, Writing – review & editing.
