# Supplementary material for: A critical systematic review assessing undergraduate neurology pipeline programs
Source: Front Med (Lausanne). 2023 Nov 21;10:1281620. doi: 10.3389/fmed.2023.1281620 (PMC10703474; doi:10.3389/fmed.2023.1281620)
Supplement: Supplementary file 1 [file Data_Sheet_1.PDF]

Appendix A: Search Strategies

PubMed

(Neurology[Mesh] OR Neurology[tiab] OR Neurosciences[Mesh] OR Neuroscience[tiab] OR Neurosciences[tiab]) AND ("primary school"[tiab] OR "elementary school"[tiab] OR "middle school"[tiab] OR "grammar school"[tiab] OR "secondary school"[tiab] OR "high school"[tiab] OR college[tiab] OR undergraduate[tiab] OR Universities[Mesh]) AND (Neurology/education[Mesh] OR Neurosciences/education[Mesh] OR pipeline[tiab] OR "education pipeline"[tiab] OR "career pipeline"[tiab] OR "pipeline program"[tiab] OR "summer program"[tiab] OR Career Choice[Mesh] OR Career[tiab] OR Education[Mesh] OR Education[tiab] OR Learning[Mesh] OR Learning[tiab] OR Motivation[Mesh] OR Motivation[tiab] OR outreach[tiab] OR Curriculum[Mesh] OR Curriculum[tiab] OR Curricula[tiab] OR extracurricular[tiab])

1070 Results – 7/5/2022

EMBASE

(*neurology/ or *neuroscience/ or (neurology or neuroscience or neurosciences).ti, ab.) and (*primary school/ or *middle school/ or *high school/ or *college/ or *undergraduate education/ or *university/ or ("primary school" or "elementary school" or "middle school" or "grammar school" or "secondary school" or "high school" or college or undergraduate). ti, ab.) and (*career/ or *education/ or *learning/ or *motivation/ or *curriculum/ or (pipeline or "education pipeline" or "career pipeline" or "pipeline program" or "summer program" or "career choice" or career or education or learning or motivation or outreach or curriculum or curricula or extracurricular).ti, ab.)

902 results – 7/5/2022

PsycINFO

(*neurology/ or *neurosciences/ or (neurology or neuroscience or neurosciences).ti,ab.) and (*Elementary Schools/ or *Middle Schools/ or *Secondary Education/ or *High Schools/ or *Colleges/ or ("primary

school" or "elementary school" or "grammar school" or "secondary school" or "high school" or college or undergraduate).ti, ab.) and (*Occupational Choice/ or *Occupations/ or *Education/ or *Learning/ or *Motivation/ or *Curriculum/ or *Educational Programs/ or (pipeline or "educational pipeline" or "career pipeline" or "pipeline program" or "summer program" or "career choice" or career or education or learning or outreach or curriculum or curricula or extracurricular).ti, ab.)

502 results – 7/5/2022

Education Source + ERIC

(DE "Neurology" OR TI neurology OR AB neurology OR DE "Neurosciences" OR TI neuroscience OR AB neuroscience OR TI neurosciences OR AB neurosciences) AND (DE "Primary schools" OR TI "primary school" OR AB "primary school" OR DE "Elementary Schools" OR TI "elementary school" OR AB "elementary school" OR DE "Middle schools" OR TI "middle school" OR AB "middle school" OR TI "grammar school" OR AB "grammar school" OR DE "Secondary schools" OR TI "secondary school" OR AB "secondary school" OR DE "High schools" OR TI "high school" OR AB "high school" OR DE "Universities & colleges" OR TI "college" OR AB "college" OR DE "Undergraduates" OR TI undergraduate OR AB undergraduate) AND (DE "Neurology education" OR TI pipeline OR AB pipeline OR TI "education pipeline" OR AB "education pipeline" OR TI "career pipeline" OR AB "career pipeline" OR TI "pipeline program" OR AB "pipeline program" OR TI "summer program" OR AB "summer program" OR DE "Summer Programs" OR DE "Career Choice" OR TI "career choice" OR AB "career choice" OR DE "Occupational Aspiration" OR TI career OR AB career OR DE "Education" OR TI education OR AB education OR DE "Learning" OR TI learning OR AB learning OR DE "Motivation" OR TI Motivation OR AB Motivation OR TI outreach OR AB outreach OR DE "Curriculum" OR TI Curriculum OR AB Curriculum OR TI Curricula OR AB Curricula OR TI extracurricular OR AB extracurricular)

418 results – 7/5/2022

378 with duplicates removed
